# Intercomparison of Small Unmanned Aircraft System (sUAS) Measurements for Atmospheric Science during the LAPSE-RATE Campaign

**DOI:** 10.3390/s19092179

**Published:** 2019-05-10

**Authors:** Lindsay Barbieri, Stephan T. Kral, Sean C. C. Bailey, Amy E. Frazier, Jamey D. Jacob, Joachim Reuder, David Brus, Phillip B. Chilson, Christopher Crick, Carrick Detweiler, Abhiram Doddi, Jack Elston, Hosein Foroutan, Javier González-Rocha, Brian R. Greene, Marcelo I. Guzman, Adam L. Houston, Ashraful Islam, Osku Kemppinen, Dale Lawrence, Elizabeth A. Pillar-Little, Shane D. Ross, Michael P. Sama, David G. Schmale, Travis J. Schuyler, Ajay Shankar, Suzanne W. Smith, Sean Waugh, Cory Dixon, Steve Borenstein, Gijs de Boer

**Affiliations:** 1 Rubenstein School of Environment and Natural Resources and Gund Insitute for Environment, University of Vermont, Burlington, VT 05401, USA; 2 Geophysical Institute and Bjerknes Centre for Climate Research, University of Bergen, Postbox 7803, 5020 Bergen, Norway; stephan.kral@uib.no (S.T.K.); joachim.reuder@uib.no (J.R.); 3 Department of Mechanical Engineering, University of Kentucky, Lexington, KY 40506, USA; sean.bailey@uky.edu (S.C.C.B.); suzanne.smith@uky.edu (S.W.S.); 4 School of Geographical Sciences and Urban Planning, Arizona State University, Tempe, AZ 85281, USA; Amy.Frazier@asu.edu; 5 Unmanned Systems Research Institute and School of Aerospace Engineering, Oklahoma State University, Stillwater, OK 74078, USA; jdjacob@okstate.edu; 6 Finnish Meteorological Institute, Erik Palménin aukio 1, P.O. Box 503, FIN-00100 Helsinki, Finland; david.brus@fmi.fi; 7 School of Meteorology, Advanced Radar Research Center, and Center for Autonomous Sensing and Sampling, University of Oklahoma, Norman, OK 73071, USA; chilson@ou.edu (P.B.C.); brian.greene@ou.edu (B.R.G.); epillarlittle@ou.edu (E.A.P.-L.); 8 Department of Computer Science, Oklahoma State University, Stillwater, OK 74078, USA; chriscrick@cs.okstate.edu; 9 Department of Computer Science and Engineering, University of Nebraska–Lincoln, Lincoln, NE 68588, USA; carrick@cse.unl.edu (C.D.); ashankar@cse.unl.edu (A.S.); 10 Department of Aerospace Engineering, University of Colorado, Boulder, CO 80309, USA; Abhiram.Doddi@colorado.edu (A.D.); dale.lawrence@colorado.edu (D.L.); 11 Black Swift Technologies, Boulder, CO 80301, USA; elstonj@blackswifttech.com; 12 Department of Civil and Environmental Engineering, Virginia Tech, Blacksburg, VA 24061, USA; hosein@vt.edu; 13 Department of Aerospace and Ocean Engineering, Virginia Tech, Blacksburg, VA 24061, USA; javig86@vt.edu; 14 Department of Chemistry, University of Kentucky, Lexington, KY 40506, USA; marcelo.guzman@uky.edu (M.I.G.); travis.schuyler@uky.edu (T.J.S.); 15 Department of Earth and Atmospheric Sciences, University of Nebraska–Lincoln, Bessey Hall 126, Lincoln, NE 68588, USA; ahouston2@unl.edu; 16 Department of Mechanical and Materials Engineering, University of Nebraska–Lincoln, Lincoln, NE 68588, USA; mislam@huskers.unl.edu; 17 Department of Physics, Kansas State University, 1228 N. 17th St., Manhattan, KS 66506, USA; okemppin@phys.ksu.edu; 18 Department of Biomedical Engineering and Mechanics, Virginia Tech, Blacksburg, VA 24061, USA; sdross@vt.edu; 19 Department of Biosystems and Agricultural Engineering, College of Agriculture, Food and Environment, University of Kentucky, Lexington, KY 40546, USA; michael.sama@uky.edu; 20 School of Plant and Environmental Sciences, Virginia Tech, Blacksburg, VA 24061, USA; dschmale@vt.edu; 21 NOAA National Severe Storms Laboratory, 120 David L. Boren Blvd., Norman, OK 73072, USA; sean.waugh@noaa.gov; 22 Integrated Remote and In Situ Sensing Program, University of Colorado, Boulder, CO 80309, USA; cory.dixon@colorado.edu (C.D.); steve.borenstein@colorado.edu (S.B.); 23 Cooperative Institute for Research in Environmental Sciences, University of Colorado, Boulder, CO 80309, USA; gijs.deboer@colorado.edu; 24 NOAA Physical Sciences Division, Boulder, CO 80305, USA

**Keywords:** sUAS, unmanned aircraft systems, unmanned aerial vehicles, UAV, sensor intercomparison, atmospheric measurements

## Abstract

Small unmanned aircraft systems (sUAS) are rapidly transforming atmospheric research. With the advancement of the development and application of these systems, improving knowledge of best practices for accurate measurement is critical for achieving scientific goals. We present results from an intercomparison of atmospheric measurement data from the Lower Atmospheric Process Studies at Elevation—a Remotely piloted Aircraft Team Experiment (LAPSE-RATE) field campaign. We evaluate a total of 38 individual sUAS with 23 unique sensor and platform configurations using a meteorological tower for reference measurements. We assess precision, bias, and time response of sUAS measurements of temperature, humidity, pressure, wind speed, and wind direction. Most sUAS measurements show broad agreement with the reference, particularly temperature and wind speed, with mean value differences of 1.6 $\pm 2.6{\;}^{\circ }$C and 0.22 ±0.59 m/s for all sUAS, respectively. sUAS platform and sensor configurations were found to contribute significantly to measurement accuracy. Sensor configurations, which included proper aspiration and radiation shielding of sensors, were found to provide the most accurate thermodynamic measurements (temperature and relative humidity), whereas sonic anemometers on multirotor platforms provided the most accurate wind measurements (horizontal speed and direction). We contribute both a characterization and assessment of sUAS for measuring atmospheric parameters, and identify important challenges and opportunities for improving scientific measurements with sUAS.

## 1. Introduction

Small unmanned aircraft systems (sUAS) are transforming the paradigm of atmospheric research. Their importance for meteorological studies has been highlighted in several recent reports [1,2], and their ability to contribute high quality measurements across spatial and temporal domains is unequivocal [3]. Initial efforts to measure the atmosphere with remotely piloted aircraft began a half century ago [4,5], and activities have continued through the 20th century [6]. However, the last decade has seen a rapid increase in the rate of sUAS development and application for lower atmospheric studies due to reductions in cost of systems and sensors associated with the advancement of consumer electronics. A main benefit of sUAS is their ability to operate in airspaces or situations that are too difficult or hazardous for manned aircraft [7] such as in and around thunderstorms [8], active volcanoes [9], or chemical plumes. Since they are more maneuverable than other types of platforms, they are able to sample portions of the atmosphere that have previously been either limited in observation or inaccessible through traditional monitoring methods such as meteorological towers, weather balloons, or satellites. They also permit the capture of atmospheric variables and data at finer spatial and temporal scales compared to other measurement technologies, and often at lower cost, allowing enhanced investigations of boundary layer processes. In sum, sUAS are providing critical information on the vertical and horizontal structure and variability of the atmosphere, which in turn is spurring new areas of engineering and science related to the development, deployment, and application of these systems for atmospheric studies.

sUAS have been employed to address a range of theoretical, methodological, and applied atmospheric science research questions. In particular, they have proven instrumental for advancing boundary layer research [10,11,12,13,14,15,16,17,18,19], gas and aerosol investigations [20,21,22,23], cloud microphysics [24,25], understanding of severe storm development [8,26,27], turbulence research [28,29], and the impact of wind turbines on atmospheric structure [30,31]. From a methodological perspective, sUAS are fostering development of new methods for measurement, such as acoustic atmospheric tomography [32], and capturing the spatial structure of thermodynamic variables [33]. In response to the growing application of sUAS technology, the European Union sponsored a COST (Cooperation in Science and Technology) Action [34] to support the development of a community around sUAS use for atmospheric science in 2008. Stemming from this action, the International Society for Atmospheric Research using Remotely piloted Aircraft (ISARRA) was established. The first meeting of ISARRA was held in Palma de Mallorca, Spain in 2013, and since then meetings have been held annually in Europe and the United States.

A key factor in the development of ISARRA was the integration of synergistic community knowledge related to atmospheric sensors, best practices for integration of sensors onboard unmanned aircraft platforms, and general measurement techniques and principles when using sUAS for atmospheric sampling, as discussed above. Critical to achieving scientific goals with sUAS is ensuring accurate measurements through proper characterization of system and sensor performance. A variety of commercial off-the-shelf (COTS) and custom-built sensor payloads for sUAS have been developed specifically to measure atmospheric variables such as thermodynamics, wind velocity/direction, turbulence, gas concentrations, and aerosol properties. An overview of some of these sensors was provided in Ref. [7] and a recent community white paper [35]. In addition, scientific capability can be impacted by the sUAS itself, with both fixed-wing and multirotor sUAS platforms used. In general, fixed-wing platforms currently have an advantage over multirotor platforms in terms of endurance and payload potential, whereas multirotor platforms typically require less operator infrastructure and expertise as well as have hovering and ascent flight capabilities that cannot be matched by fixed-wing platforms. Thus, obtaining accurate measurements depends not only on the integrity of the sensor technology but also on intrinsic factors such as the manner in which the sensor is integrated into the platform, as well as extrinsic factors such as flight patterns and weather conditions. Beyond the sensors themselves, platforms can add additional uncertainties to the measurements. For example, multirotor sUAS can introduce localized mixing of the atmosphere from the propellers, which alters the environment being sampled. Additionally, the reduced forward motion of multirotor platforms compared to fixed-wing aircraft can also impact measurements by reducing airflow over sensors and contributing to directional solar effects. Studies have begun investigating the impacts of these and other factors on the quality of thermodynamic measurements [36,37], but a greater understanding of how platform and sensor characteristics affect measurement quality is needed.

**Figure 1:**
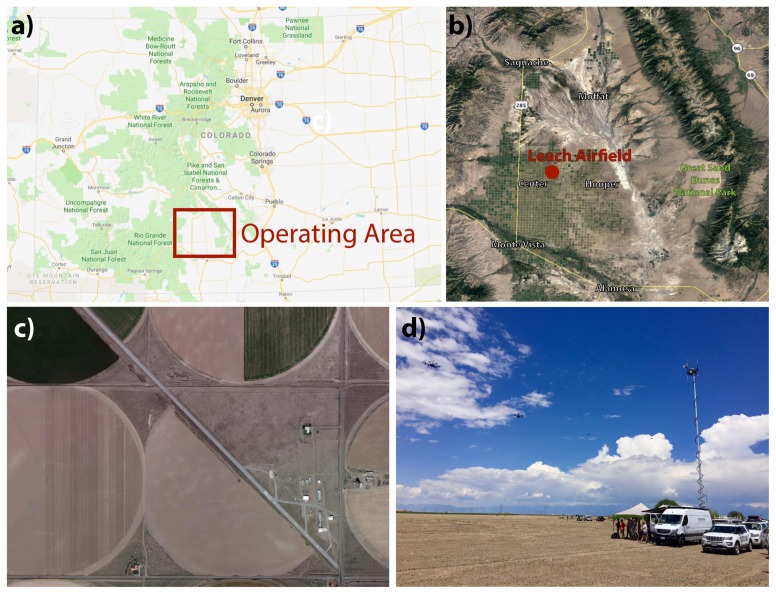
Maps illustrating the location of flight operations during Lower Atmospheric Process Studies at Elevation—a Remotely piloted Aircraft Team Experiment (LAPSE-RATE). (**a**) Operating area. (**b**) Leach Airfield. (**c**) The largest map (right) shows a satellite image of the area around Leach Airfield [Images courtesy of Google Maps]. (**d**) Mobile UAS Research Collaboratory (MURC) facility.

Given the rapid expansion in the use of sUAS for atmospheric research, there is a pressing need to continually assess and improve the quality of the instrumentation and measurement devices to advance collective understanding of the robustness of data being captured from sUAS. As part of the most recent ISARRA meeting, hosted by the University of Colorado-Boulder (CU-Boulder) during summer 2018, a community field campaign was organized in which a primary scientific goal was the characterization of system and sensor performance for improving the quality of atmospheric measurements. The field campaign, titled ‘Lower Atmospheric Process Studies at Elevation—a Remotely piloted Aircraft Team Experiment (LAPSE-RATE)’, took place in the San Luis Valley of Colorado (Figure 1) from 14–19 July, 2018, and included participation by a variety of university, government, and industry teams. Over the course of six days, more than 100 participants from 13 institutions and organizations supported the coordinated deployment of over 35 unmanned aircraft and completed 1287 flights, accumulating more than 260 flight hours. Flight operations spanned a large area of the San Luis Valley (approximately 3500 km${}^{2}$), and distributed research flights were organized to observe several interesting atmospheric phenomena, including the morning boundary layer transition in a high-altitude mountain valley, the diurnal cycle of valley flows, convective initiation, and aerosol properties.

In addition to these scientific objectives, coordinated missions were organized between the participating teams to compare measurements across sensors and platforms and validate these measurements against reference measurements from ground-based instrumentation, in particular from a 18 m meteorological tower. The purpose of these intercomparisons was to not only compare the performance of different sensors to the ground-based references but also to compare sensor performance across platforms. The objectives of this study were to evaluate the measurements collected during intercomparison flights to provide a deeper understanding of the accuracy related to capturing atmospheric measurements via sUAS and to identify factors that may contribute uncertainties or error to meteorological measurements. In the next section, Section 2, we provide an overview of the site information, a detailed description of the sUAS and ground-based systems used for intercomparison as well as information on the flight patterns and analysis techniques. Section 3 presents the results of these comparisons including evaluation of intercomparisons between sUAS-based measurements and ground-based measurements as well as statistical comparisons between measurements captured by different aircraft systems. In addition, this section discusses these intercomparison results in detail and reflects on the potential causes of the observed differences, best practices based on the results of this intercomparison study, and additional perspectives on the future direction of sUAS-based atmospheric measurement.

## 2. Materials and Methods

### 2.1. Study Site Information

#### 2.1.1. Operations Area and Ground Instrumentation

The comprehensive LAPSE-RATE campaign took place across the northern half of the San Luis Valley, but all intercomparison flights analyzed in this study were conducted at the Leach Airfield (37${}^{\circ }$47${}^{\prime }$06${}^{\prime \prime }$ N 106${}^{\circ }$02${}^{\prime }$49${}^{\prime \prime }$ W) located in Center, Colorado (Figure 1). The local time during this study at this site was MDT, and all times reported in this study are in UTC, which is +8 h from MDT. The airfield is a county-owned, public-use airport located approximately 3.2 km ENE of the commercial district of Center, Colorado and 32 km NNW of Alamosa, Colorado. The site is situated approximately 2330 m above sea level (MSL) and, true to its name, sits in the center of the expansive elevated San Luis valley. The airfield is surrounded by irrigated agricultural land with very little topography in the immediate vicinity, although substantial mountain ranges (some peaks over 4300 m) are located approximately 32 km to the east and west, 40 km to the north, and 112 km to the south. The open space around Leach Airfield supported simultaneous deployment of several sUAS at a time.

The Mobile UAS Research Collaboratory (MURC) was the primary ground-based system providing instrumentation for reference comparisons. The MURC is an instrumented van that was added to CU-Boulder’s Integrated Remote and In-Situ Sensing (IRISS) program vehicle fleet in early 2018 (Figure 1d). The MURC was designed to operate independently during sUAS operations and serve as a mobile command station on larger deployments, overseeing field teams and providing situational awareness. The MURC is equipped with a 15 m extendable mast, at the top of which several meteorological sensors are mounted (Figure 1d). These include a Gill MetPak Pro Base Station that provides barometric pressure (±0.5 hPa), temperature ($\pm 0.1{\;}^{\circ }$C), and humidity (±0.8% of RH); a Gill 3D sonic anemometer (<1.5% RMS accuracy at 12 m/s, $\pm 2^{\circ }$ accuracy at 12 m/s) for 3D wind measurements; and an R.M. Young Wind Monitor anemometer (±0.3 m/s, $\pm 3^{\circ }$), which provides a redundant horizontal wind measurement. All meteorological sensors were purchased and installed a few months prior to the LAPSE-RATE campaign, and instrument accuracy is provided from the manufacturers specifications. All together termed the MURC Tower, the vehicle and mast with this instrumentation was 18 m tall. The MURC also contained a large communications suite that increases the range of the UHF/VHF vehicle to vehicle radios, increases bandwidth on the cellular data connection, and improves the ground station to sUAS communication link. For field computing and campaign support, the MURC was equipped with two workstations serving as sUAS ground stations. Additionally, there were two servers for more intensive computing tasks, with one dedicated to graphics intensive processes (such as photogrammetry) and the other dedicated to general computing and data processing.

The Integrated Mesonet and Tracker (CoMet-2) was an additional mobile ground-based system that was operational during the intercomparison flights at Leach Airfield. This unit provided near-ground observations with slow temperature and humidity at ∼2 m altitude measured using a Vaisala HMP155A, pressure at 2.5 m altitude using a Vaisala PTB210, and wind at 3.25 m altitude using an R.M. Young 05103 propellor anemometer. While this ground-based system did provide important contextual atmospheric measurements, especially for the multirotor platforms, these data were not used in our primary analysis. Several additional ground-based observational assets were also deployed throughout the week, including two ground-based Doppler LiDAR (Windcube) systems operated by CU-Boulder, the Collaborative Lower Atmospheric Mobile Profiling System (CLAMPS) operated by the University of Oklahoma and the National Oceanic and Atmospheric Administration (NOAA) National Severe Storms Laboratory (NSSL), ground vehicles outfitted with meteorological sensors operated by the University of Nebraska–Lincoln and NSSL (following Ref. [38]), and regular radiosonde launches. While these data again served to better contextualize the meteorological conditions, they were not used directly in the intercomparison analyses.

The majority of the intercomparison flights were conducted on 14 July 2018, with additional intercomparison flights taking place on 15 and 17 July 2018. While it was not possible to fly all platforms simultaneously given space constraints and Federal Aviation Administration (FAA) sUAS operating procedures, all platforms were flown in similar flight patterns and at approximately the same altitude and distance from the ground instrumentation to standardize comparisons. All intercomparison flights were conducted under FAA Part 107 [39].

#### 2.1.2. Weather Conditions

**Figure 2:**
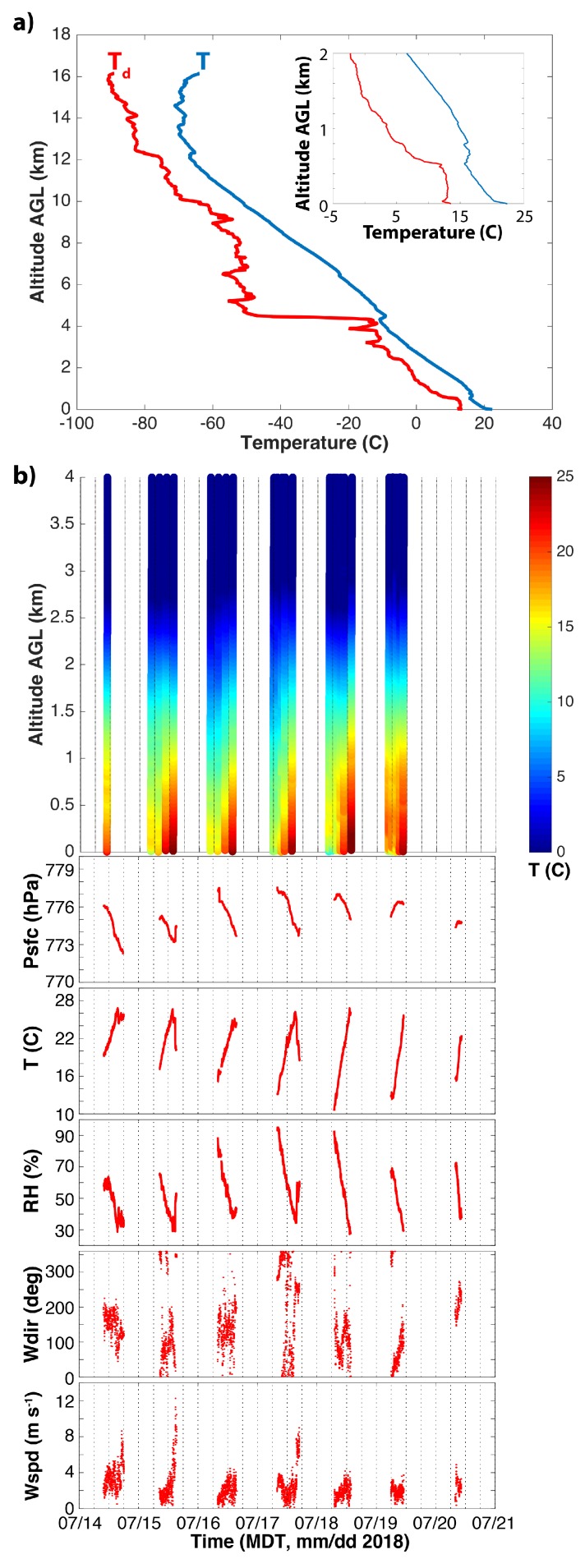
Radiosonde data. (**a**) Radiosonde data, including temperature (*T*, blue line) and dew point temperature ($T_{d}$, red line), from 14 July (17:43 UTC launch time) and (**a**)—insert focuses on the radiosonde data from surface to 2 km above ground level (AGL). (**b**) Data from radiosonde launches (top) and MURC (bottom) for each day during the week-long campaign.

Forecasting and modeling support for the entire LAPSE-RATE campaign was provided by the National Weather Service forecast office in Pueblo, CO, and the National Center for Atmospheric Research. Summer conditions in the San Luis Valley are generally dry but with frequent afternoon convection over the surrounding mountains. Some mountain storms advect over the valley itself, depending on wind and moisture conditions. Radiosonde data from the week are presented in Figure 2b, along with MURC observations of pressure (Psfc), temperature (T), relative humidity (RH), and wind speed (Wspd) and direction (Wdir) for the same time period. On 14 and 15 July, the area was impacted by a cold front to the north and monsoonal moisture advected in from the Pacific. This combination resulted in the development of widespread afternoon thunderstorms over the mountains surrounding the valley, with some storms producing heavy precipitation and gusty winds. For 17 July, a region of high pressure established over Colorado, with some storms developing over surrounding mountains. Radiosondes launched at Leach Airfield (Figure 2b) throughout the entire week of the campaign show a consistent lower atmosphere featuring a strong (15–20 ${}^{\circ }$C) diurnal cycle in temperature, with early morning temperatures around 10–12 ${}^{\circ }$C and afternoon temperatures reaching over 30 ${}^{\circ }$C. In general, winds during the intercomparison flights were light and variable, with some elevated wind speeds associated with afternoon convective events.

As stated previously, most intercomparison flights took place on 14 July 2018, with some additional flights on 15 and 17 July. A radiosonde launched on 14 July (17:43 UTC launch time, (Figure 2a) reveals a well-mixed, dry-adiabatic boundary layer extending up to around 550 m above ground level (AGL) (Figure 2a—insert), where a small temperature inversion is present. The atmosphere above this inversion layer is well-mixed up to around 4000 m AGL. The radiosonde and comparison data from surface instrumentation show the presence of a super-adiabatic surface layer extending to nearly 40 m AGL. As a result, there were generally warm and clear conditions throughout the morning and early afternoon hours. Thunderstorms formed over adjacent mountains during the afternoon, with the largest storms developing over the Sangre de Cristo range to the east of Leach Airfield (Figure 1). Some of these storms advected over the valley throughout the course of the late afternoon, resulting in gusty winds and precipitation, particularly over the eastern half of the valley. All intercomparison flights were carried out during conditions with a well-mixed lower atmosphere over the extent of the altitudes sampled.

### 2.2. sUAS Platforms, Payloads and Flight Patterns

The teams participating in the LAPSE-RATE campaign aimed to explore a wide variety of scientific objectives throughout the week, and thus there was a variety of platforms and sensors included in the intercomparison. A total of 37 individual platforms were flown including 14 different airframes and 23 unique configurations of airframes and sensor payloads. The majority of the platforms (27) were multirotor airframes, and the remaining were fixed-wing. Seventeen different types of sensors were used to measure pressure (*P*), temperature (*T*), and relative humidity (RH), and another eight different types of sensors were used to measure (horizontal) wind speed (*U*) and wind direction (dir). An overview of the different systems and operators is provided in Table 1, with more detail provided below.

#### 2.2.1. Multirotor Aircraft

The multirotor aircraft primarily consisted of COTS quadcopters (3DR SOLO, DJI Inspire 2, LynxMotion HQuad500), hexacopters (Tarot X6, DJI Matrice M600P), and octocopters (DJI S1000), with the EngeniusMicro, LLC team operating an Intense Eye V2 quadcopter manufactured by Emergent RC. The flight controllers were mainly 3DR Pixhawk, DJI proprietary, or A3 controllers. Flight times for these aircraft ranged from 12 min to 40 min, depending on their payload and rotor blade configuration.

While many of these aircraft carried sensors specific to the scientific objectives of the operating team (e.g., gas concentration, aerosols, etc.), in this study we considered only pressure, temperature, humidity, and 2D wind vector measurements. The most commonly used COTS *P*,*T*, and RH sensor was the iMetXQ/iMetXQ-2 (International Met Systems, Grand Rapids, MI, USA). These sensors log data at 1 Hz, with a stated response and accuracy of 10 ms, ±1.5 hPa for pressure; 2 s, $\pm 0.3{\;}^{\circ }$C for temperature; and 5 s, ±0.5% of RH for relative humidity for the iMetXQ and improved temperature and humidity response of 1 s and 0.6 s respectively for the iMetXQ-2. Several teams also used the Bosch BME280 sensor, which has a manufacturer-stated response and accuracy of 6 ms, ±1 hPa for pressure, 1 s, $\pm 0.5{\;}^{\circ }$C for temperature, and 1 s, ±3% of RH for relative humidity. Additional *P*, *T*, and *H* sensing on the Finnish Meteorological Institute (FMI) aircraft was also provided by a Vaisala AQT400 gas sensor, which has a manufacturer-stated accuracy of ±10 hPa for pressure, $\pm 0.3{\;}^{\circ }$C for temperature, and ±5% to 8% of RH for relative humidity and manufacturer-stated response time <60 s. It is important to note that there are nuances and limitations to manufacturer-stated sensor specifications, for example, humidity response time is dependent on temperature (e.g., for the iMetXQ-2 humidity response is 0.6 s at 25 ${}^{\circ }$C, but 5.2 s at 5 ${}^{\circ }$C), and this information is not always provided in the manufacturer specifications for all sensors.

Many of the teams participating in the LAPSE-RATE field campaign also developed their own integrated systems for meteorological measurements. For example, the University of Oklahoma (OU) operated the LynxMotion HQuad500 (CopterSonde 2), which was equipped with three Innovative Sensor Technology HYT 271 humidity sensors, three InterMet Bead Thermistors, and an TE Connectivity MS-5611 Barometer to measure pressure (8 ms, ±1.5 hPa for pressure, 1 s, $\pm 0.3{\;}^{\circ }$C for temperature, 4 s and ±1.8% of RH for humidity). This platform also utilized aircraft dynamics to extract wind speed and direction. The University of Nebraska–Lincoln (UNL) fielded a custom-built two-node pressure, temperature and humidity sensor, here referred to as the Nimbus PTH sensor. Similarly, Oklahoma State University developed the MDASS, Meteorological Data Acquisition Sonde System, to measure *P*, *T*, and RH, in addition to other user defined parameters such as GPS, radiation, wind speed, or turbulence [40]. This modular sensor was flown on several of their platforms as well as onboard the DJI M600Pro operated by Kansas State University. The OSU MDASS system has a reported accuracy of $\pm 0.3{\;}^{\circ }$C, ±2% of RH and ±0.12 hPa, an onboard fan for sensor aspiration, and shielding. The Intense Eye V2 operated by EngeniusMicro, LLC was equipped with a Differential Temperature Sensor System V2 Low Mass Flex to measure temperature at a resolution of 0.00625 ${}^{\circ }$C and also included a TriSonica Mini Weather Station to measure pressure, temperature, and humidity (±3 hPa, $\pm 0.5{\;}^{\circ }$C, ±3% of RH), and also wind speed and direction.

Several multirotors utilized sonic anemometers for measuring wind speed and direction [41]. Sonic anemometers used included the Trisonica Mini (±0.5 m/s magnitude, $\pm 1^{\circ }$ direction, $\pm 2{\;}^{\circ }$C temperature), FT Technologies FT205 (±0.3 m/s magnitude, $\pm 4^{\circ }$ direction, $\pm 2{\;}^{\circ }$C temperature), Meter Atmos 22 (±0.5 m/s magnitude, $\pm 1^{\circ }$ direction) [41], and R.M. Young 81000 (±0.05 m/s magnitude, $\pm 2^{\circ }$ direction, $\pm 2{\;}^{\circ }$C, temperature. All sonic anemometers used in this study were located on masts above the plane of the rotors to avoid rotor wash effects. In addition, these sensors also provide a temperature measurement capability, which is included in this study. However, it should be noted that this sonic temperature is an inferred value that is also influenced by humidity, and is not a direct temperature measurement, but very close to virtual temperature.

Wind velocity estimates from the motion of an sUAS platform in flight can also be obtained either by using a kinematic or dynamic model [42,43,44,45,46]. Kinematic models are used to measure wind velocity solely from a sUAS platform orientation obtained from IMU measurements, whereas dynamic models consider the flight dynamics of the sUAS platform (i.e., how forces and moments relate to vehicle accelerations) in addition IMU measurements to estimate the wind. There are advantages and disadvantages to each with primary limitations arising from IMU accuracy and vehicle inertial response times [47].

#### 2.2.2. Fixed-Wing Aircraft

The fixed-wing aircraft included a combination of modified, COTS, and custom airframes. CU-Boulder (CU) operated four types of fixed-wing aircraft: TTwistor-3, Talon-3, Mistral, and Datahawk2. The TTwistor is an update to the field proven Tempest sUAS but with increased performance [48,49]. The TTwistor-3 and Mistral airframes are made from composites, while the DataHawk2 and Talon-3 are foam construction. TTwistor-3 has an endurance of up to 3 h at 17 m/s, the Mistral’s endurance is about 2 h, the DataHawk2’s is about 60 min, and the Talon’s is about 30–45 min. The TTwistor-3, Mistral, and Talon-3 aircraft make use of the Pixhawk autopilot, while the Datahawk2 employs custom-developed avionics software. TTwistor-3 is equipped with a PTU module that is based upon the sensors employed by the National Center for Atmospheric Research (NCAR) mini Dropsonde. This system uses a Vaisala RSS904 sensor module, which is near identical to the same sensors used in the standard RS-92 radiosonde [50] except for the temperature sensor, which is a larger and more mechanically robust sensor. Additionally, TTwistor-3 carries an Aeroprobe 5-hole multi-hole probe [51,52] for 3D relative wind measurements; and a Vectornav VN200 [53,54] for position and orientation. The Mistral is equipped with a BlackSwift Technologies multi-hole probe (MHP), which provides 3D relative wind along with *P*, *T*, and RH. Talon-3 is also equipped with the Vaisala PTU module under one wing and a Microsonde board under the other. The Microsonde board has a TE connectivity MS-8607 *P*, *T*, and RH sensor, [55], along with a uBlox Global Navigation Satellite System (GNSS) module [56].

The Datahawk2 sUAS, [57] is a small pusher-prop foam aircraft. This platform has been used for a variety of purposes, including the study of turbulence [58,59] and high latitude [10,23] deployments. The DataHawk2 carries a variety of sensors to make measurements of the atmospheric and surface states. Custom instrumentation includes a fine wire sensor employing two cold-wires and one hot-wire. These provide high frequency (800 Hz) information on temperature and wind speed. High bandwidth is enabled by small surface-area-to-volume ratios of very thin (5 μm diameter) wires. In addition, the DataHawk2 carries a custom integrated circuit that includes commercial Sensiron SHT-31 sensor for temperature and humidity (±1.5% of RH, $\pm 0.1{\;}^{\circ }$C, 8 s response time) and a TE Connectivity MS-5611 for barometric pressure (±1.5 hPa, 8 ms response time). For information on surface and sky temperatures, DataHawk2s are also equipped with upward- and downward-looking thermopile sensors. Wind speed and direction is determined from the measured ground velocity vector, aircraft attitude, and measured air speed relative to the aircraft.

Black Swift Technologies (BST) worked with CU-Boulder (CU) to operate two additional fixed-wing aircraft, the S1 and S2. The S1 is a foam airframe based on the commercially available Skywalker X8 platform and outfitted with the Black Swift Technologies SwiftPilot autopilot. It has a flying-wing design with a 2.1 m wingspan and gross takeoff weight of 5 kg, and 0.5 kg available for carrying sensors. In the S1 configuration, the aircraft can operate for up to 90 min at a cruise speed of 15 m/s. The S2 is a fully composite airframe purpose-built for flying scientific payloads in demanding atmospheric environments (high-altitude, corrosive particulates, and strong turbulence). It is also outfitted with the SwiftCore autopilot system. The aircraft has a maximum take off weight of 8.1 kg and a wingspan of 3.0 m, providing an 18 m/s cruise speed for up to 110 min. Both the S1 and S2 were equipped with the BST multi-hole probe which provides wind speed, direction, magnetometer, accelerometer, gyroscope, barometric pressure, temperature and humidity measurements at 100 Hz. The sensors were placed on the top of the aircraft, with the probe tip extending beyond the front of the aircraft to reduce body effects on the probe measurements. State measurements from the SwiftCore autopilot were used to provide the necessary information for conversion of the wind vectors to the inertial frame.

The University of Kentucky (UKY) operated three fixed-wing aircraft, which are virtually identical and are referred to as the BLUECAT5 design [29]. These aircraft were also built around the Skywalker X8 airframe, ruggedized and modified for autonomous flight using a 3DR Pixhawk PX4 autopilot. In their current configuration, the aircraft can operate for 45 min at a cruise speed of 20 m/s. Each BLUECAT5 was equipped with an iMet-XQ sensor to measure *P*, *T*, and RH. The sensor was located on top of the aircraft fuselage in a housing designed to protect the sensor from solar radiation, while also leaving it exposed to air flowing over the aircraft. Wind velocity and direction were determined by a custom five-hole probe to measure the air velocity vector relative to the aircraft, working with a VectorNav VN-300 dual GNSS inertial sensor to measure the aircraft ground speed and aircraft orientation in the Earth-fixed inertial frame.

#### 2.2.3. Sensor Locations

Sensor location varied for each aircraft with each team designing their own solutions. For simplicity, we assign them to the following categories: (1) indicates systems where *T* and RH sensors are not impacted by solar radiation shielding or forced aspiration; (2) indicates no special sensor placement, aspiration, or solar radiation protection for *T*, and RH sensors; (3) indicates aspiration and solar shielding for *T*, and RH sensors; (4) indicates system has solar shielding but no forced aspiration for *T*, and RH sensors; and (5) indicates system with aspiration but no solar shielding for *T* and RH sensors. Generally speaking, forced aspiration is assumed for all fixed-wing aircraft, as the sensors were located external to the airframe. The most common configuration for aspirating the multirotor sensors was by placing them within the rotor wash. Approaches to solar shielding *T* and RH sensors varied considerably by aircraft.

#### 2.2.4. Flight Patterns

**Figure 3:**
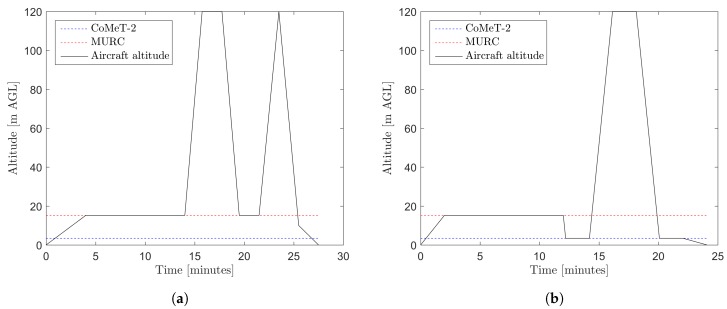
Example flight profiles. (**a**) Fixed-Wing; (**b**) Rotorcraft.

All sUAS platforms with payload packages conducted their intercomparison flights under the guidance of pre-defined flight patterns. The two standard patterns for fixed-wing and multirotor platforms are described below and visualized in Figure 3. Pilots adhered to those patterns as closely as possible, and any deviations that occurred during individual intercomparison flights, e.g., due to operational limitations, were recorded.

The primary comparison measurements were extracted from flight loiters conducted at approximately 18 m, to match the height of the MURC. In some cases, particularly for fixed-wing platforms, 18 m was too low for safe flight operations and slightly higher altitudes were chosen. Pilots loitered for 10 min to stabilize and obtain equilibrated sensor measurements for comparison with MURC. In the case of the multirotor platforms, an additional loiter at 3.4 m was performed to match with CoMeT-2. If sUAS battery operations or other flight conditions did not allow for the full 10 min, then 8 min was acceptable for loiters with the MURC for the primary time of measurement comparison, and 2 min for other altitudes. In addition to loiters, sUAS platforms ascended to 120 m and descended through the altitude point of 18 m to correspond with MURC tower heights to compare lag effects of sUAS sensor measurements. Flight patterns were conducted at least once for every sUAS platform and sensor payload. Specifically, the nominal flight patterns were as follows. *Fixed-Wing*: Launch and fly to stabilize at loiter altitude. Loiter at 18 m for 10 min. Ascend at 1 m/s to 120 m. Loiter at 120 m for 2 min. Descend 1 m/s to 18 m. Loiter at 18 m for 2 min. Ascend at own rate to 120 m. Descend at own rate to pass through 18 m. Land. *Multirotor*: Launch and fly to stabilize at loiter altitude. Loiter at 18 m for 8–10 min. Descend 1 m/s to 3.4 m. Loiter 3.4 for 2 min. Ascend at 1 m/s to pass through 18 m to reach 120 m. Loiter at 120 m for 2 min (as battery allows). Descend at 1 m/s to pass through 18 m to reach 3.4 m. Loiter 2 min at 3.4 m (as battery allows). Land.

### 2.3. Data Analysis

As noted above, we grouped the sUAS platforms with sensor payloads by the platform configuration (fixed-wing or multirotor) and the sensor placement configuration. We then analyzed each sUAS platform and sensor payload by each atmospheric parameter measured: temperature, relative humidity, pressure, wind speed, wind direction. For each atmospheric parameter, we compared the sUAS measurements with the MURC reference measurements during sUAS flight loiter time. In some cases it was hard to identify a time period during the loiter where clear steady-state sensor values had been reached. In such cases we selected a intercomparison period covering roughly the second half of the loiter period. Comparisons of overall time series were made and a measurement difference plot was produced using the mean values from the intercomparison period. A Wilcoxon rank-sum test, also known as a Mann–Whitney U, test was then performed on sUAS mean measurement differences from the MURC reference to test for significant differences between (a) aircraft platform type (fixed-wing and multirotor) and (b) sensor configurations. These tests were repeated for all parameters.

Due to the fact that the analyzed parameters may vary significantly with altitude and since the algorithms for determining of the flight altitude may be very different for the sUAS in use, we applied a uniform post-processing algorithm to determine the flight altitude from pressure and temperature data. For the sUAS not sampling both of these parameters the originally provided altitude, mostly GNSS based, was used. In the applied method we first detrend the pressure time series linearly based on the pressure just before the start and right after the landing to remove a potential sensor drift or temporal atmospheric changes. We then compute the vertical thickness between two adjacent pressure levels of Δp=0.5 hPa increments:

(1) $$\Delta z_{i}=-\frac{\Delta p}{g\cdot \rho_{i}}$$

with the density, $\rho_{i}=\bar{p_{i}}/R\cdot \bar{T_{i}}$, calculated based on the mean pressure, $\bar{p_{i}}$, and mean temperature, $\bar{T_{i}}$, of each layer *i* and the specific gas constant of dry air, R=287.058 J kg${}^{-1}$ K${}^{-1}$. The integration of $\Delta z_{i}$ yields an altitude $z_{i}$ for each pressure level $p_{i}$. By interpolating between the pressure levels $p_{i}$ for each p(t) we get a new time vector z(t). Apart from improving the comparability of our data, to our experience this method usually gives a much more stable altitude than the pure GNSS reading and assures that start and landing are at the same level. Furthermore, taking the ambient temperature into account improves the accuracy under conditions deviating significantly from the typically assumed standard atmosphere profile.

To compare sUAS measurements to the reference MURC data, precision and bias analysis was constrained to data from the portion of the flight when the sUAS was at the same altitude as the MURC (approximately 10 min). As sensors had variable response times, with some responding more slowly upon reaching loiter altitude, we further constrained the measurement data to the period of time in which parameter measurements were determined to be stable and in equilibrium. Further, it is important to note that many wind estimates from these platforms require extensive post-processing.

To assess time response we compared the ascent and descent portion of the flight profile. We base our analysis on the assumption that the mean profile was stationary during this portion of the flight paired with using concurrent MURC data as a reference, and that therefore large statistical deviations between the profiles measured during ascent and descent could be attributed solely to lag caused by insufficient measurement system time response, with increasing deviation reflecting increased time lag. Note that this difference is influenced by the sensor itself, as well as the intricacies of the sensor placement on the aircraft. Thus, the profile data was split into ascent and descent portions of the flight using visual inspection of the altitude, temperature, humidity and pressure time series. Then, the ascent and descent data were bin-averaged using 1 m vertical bins between 19.5 m and 120.5 m. Where data were not available within these bins due to high ascent/descent rates relative to the sensor acquisition rate, linear interpolation was used to ensure at least one measurement point was present.

To generate a single, simple measure of sensor response, the absolute-mean-deviation (amd) between the bin-averaged ascent and descent data of a particular quantity, *X*, was calculated as

(2) $$amd\left(X^{↑↓}\right)=\left|{\langle X_{i}^{↑}-X_{i}^{↓}\rangle }_{i}\right|$$

where the ↑ indicates data from ascent, the ↓ indicates data from descent, the $X_{i}$ indicates the quantity measured for each altitude bin, and the ${\langle \cdot \rangle }_{i}$ brackets indicate an average over all bins. Note that averaging before taking the absolute value is important to account for the fact that fast-response sensors resolving fine-scale turbulence may produce ascent and descent profiles very close together, but the profiles may cross each other several times. By calculating the absolute-mean-deviation, positive and negative deviations may cancel each other out, leading to a low value for the entire profile. Hence, following the assumption of a stationary mean profile, a low amd value should reflect faster temporal response of the measurement system.

Since each comparison flight took place at a different time, the mean-absolute deviation (mad) from the time-series data measured by the MURC was computed to provide an indication of the natural variability of *X* that may have occurred during each profiling flight. For the portion of the MURC time-series, $X_{M}\left(t\right)$, corresponding to the ascent and descent portion of each specific sUAS flight, we calculate

(3) $$mad\left(X_{M}\right)=\langle |X_{M}\left(t\right)-{\langle X_{M}\left(t\right)\rangle }_{t}{|\rangle }_{t},$$

where $X_{M}\left(t\right)$ is the quantity measured during the portion of the time series being considered and ${\langle \cdot \rangle }_{t}$ indicates a time average over that portion of the time series. A higher mad value will indicate increasing deviation from the assumed stationary mean profile and therefore indicates that there is a contribution of non-stationarity for the amd value for a particular measurement system.

## 3. Results and Discussion

### 3.1. Overview of Precision and Bias Results

The majority of the intercomparison analyses presented here are from each sUAS intercomparison flight using the MURC data as a consistent reference. By using the measurements from when the flight altitude was at, or close to, the altitude of the MURC tower, we assume statistical convergence of measured parameters, and focus on precision and bias differences between the sUAS and the MURC measurements. The horizontal separation between the sUAS and the MURC, in the order of 15 m for multirotors and typically 100 m for fixed-wings, is assumed to be insignificant with respect to the averaging times and the prevailing wind speeds. However, it has to be noted that averaging fixed-wing data, which are sampled along a circular trajectory, implies spatial averaging as well which is not the case for stationary platforms such as the MURC tower or a loitering multirotor system.

**Figure 4:**
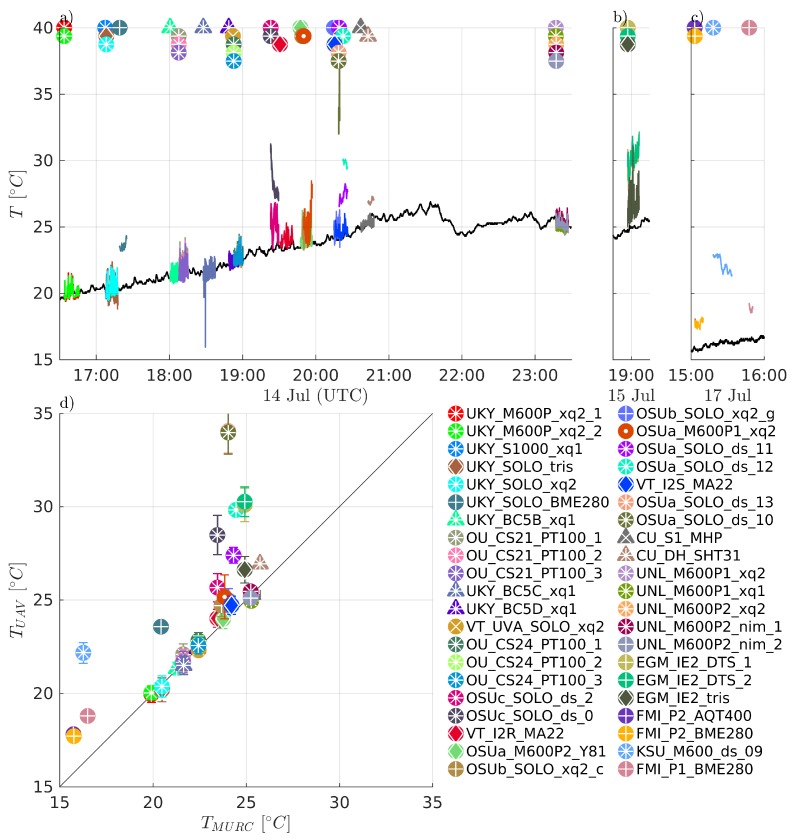
Time series of temperature measurements during July 14 (**a**), 15 (**b**), and 17 (**c**). The black solid line shows the reference observations from the MURC at 18 m AGL, whereas the colored solid lines represent the small unmanned aircraft system (sUAS) observations from the comparison period when the sUAS was flying next to the tower at approximately the same height. The colored symbols at the top mark the start time of the comparison period and their shape indicates the type of sUAS: △ for fixed-wing systems, and a ◯ for multirotor systems. The different white markers on top of the colored symbols indicate the different types of *T* and relative humidity (RH) sensor setups: · for no aspiration and radiation shielding; × for aspiration only; + for radiation shielding only; * for aspiration and radiation shielding; ⋄ for sensors not impacted by aspiration or solar radiation shielding. The mean differences between the sUAS and the MURC reference observations are shown in (**d**) with error bars indicating standard deviation.

**Figure 5:**
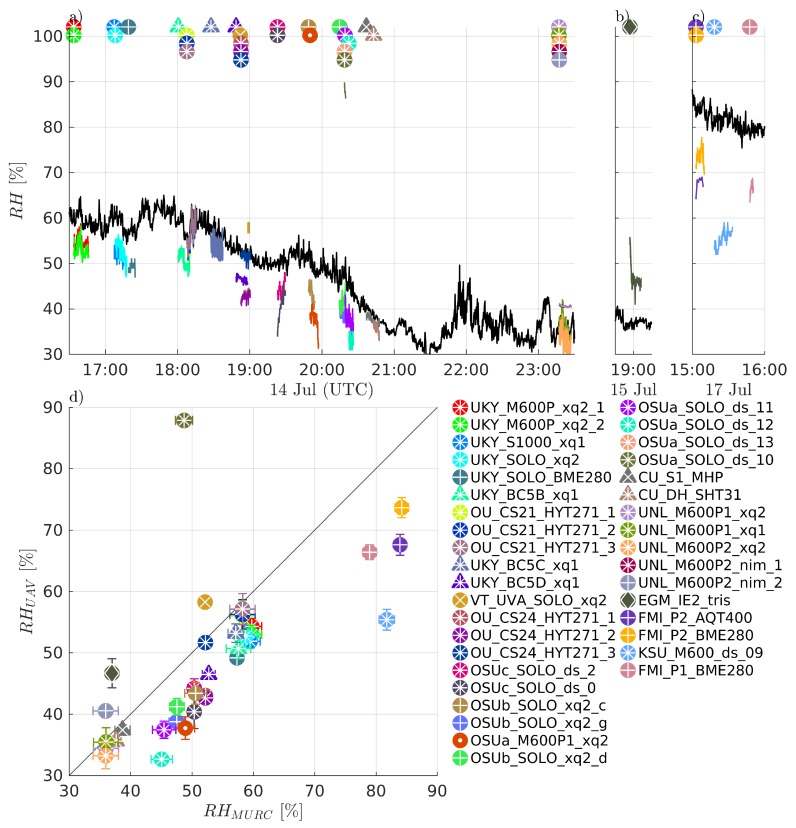
Time series of relative humidity measurements from July 14 (**a**), 15 (**b**), and 17 (**c**) and comparison of mean values to MURC reference measurements (**d**). The colors and markers follow the same scheme as in Figure 4.

**Figure 6:**
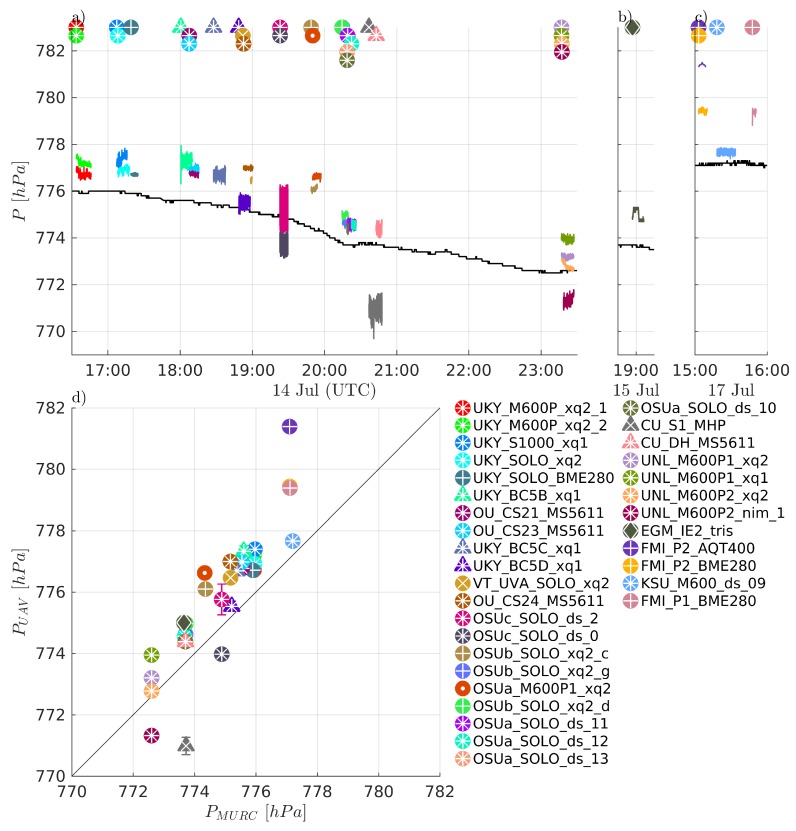
Time series of pressure measurements during July 14 (**a**), 15 (**b**), and 17 (**c**) and differences to MURC reference measurements (**d**). The colors and markers follow the same scheme as in Figure 4.

**Figure 7:**
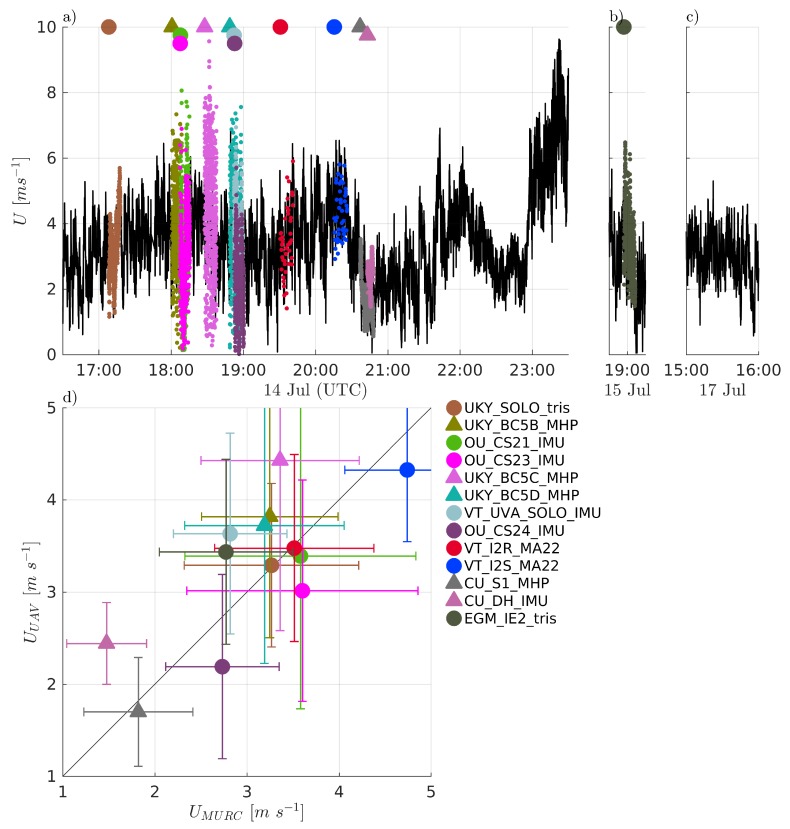
Time series of wind speed measurements during July 14 (**a**), 15 (**b**), and 17 (**c**) and differences to MURC reference measurements (**d**). The colors and markers follow the same scheme as in Figure 4, although without indication of the sensor setup (white markers).

**Figure 8:**
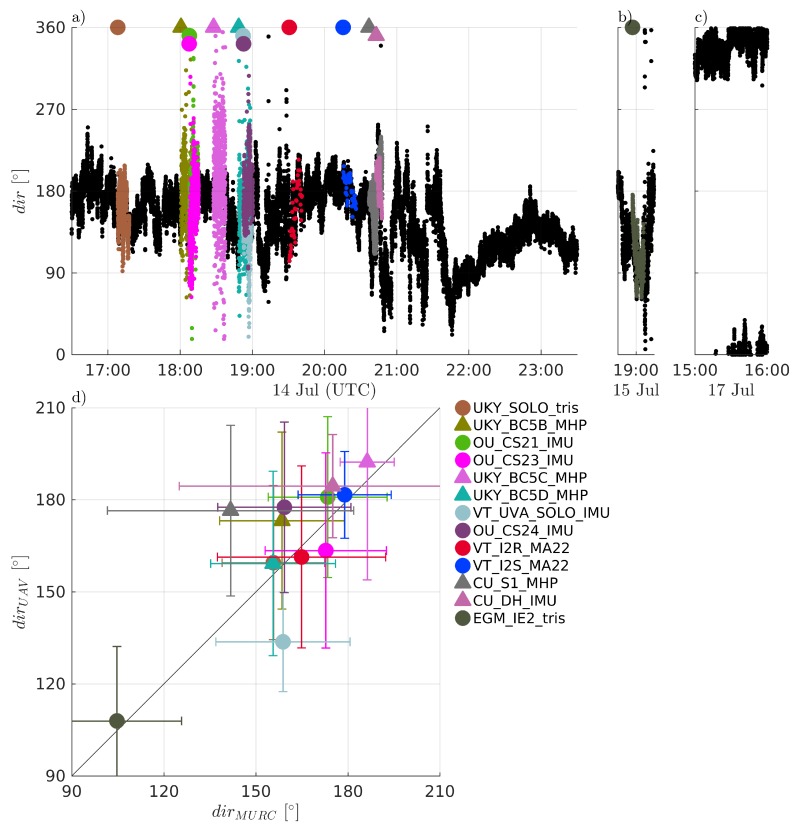
Time series of wind direction measurements (represented as dots for better interpretability) during July 14 (**a**), 15 (**b**), and 17 (**c**) and differences to MURC reference measurements (**d**). The colors and markers follow the same scheme as in Figure 7.

Comparisons of measured temperature, *T*, humidity RH, and pressure *P* are presented in Figure 4, Figure 5 and Figure 6, respectively. Humidity is presented as relative humidity, RH, as this is the common sensor output quantity for RH. Comparisons of measured (horizontal) wind speed, *U*, and direction, dir, are also presented in Figure 7 and Figure 8, respectively. In Figure 4 through Figure 8, the comparison is presented in two ways. Subfigures (a) through (c) show time series of the corresponding MURC measured parameter and the different sUAS measurements of the same parameter during the intercomparison period. Here, the solid black line represents the reference data from the MURC, and the solid colored lines reference the corresponding data from the sUAS platforms. The colored symbols at the top mark the start time of the intercomparison time period and identify the sUAS conducting flight operations. In subfigure (d), the mean value over the intercomparison time period measured by the sUAS is compared to the mean value measured by the MURC for the same intercomparison time period. Error bars in (d) represent the standard deviation of the measured values over the same time period. In Figure 4, Figure 5, Figure 6, Figure 7 and Figure 8, the same symbol nomenclature is used for consistency. In this nomenclature, a △ is used to indicate fixed-wing systems, and a ◯ is used to indicate multirotor systems. The *T* and RH sensor configurations are further indicated by additional white markers on top of the colored symbols: • for no aspiration and radiation shielding; × for aspiration only; + for radiation shielding only; ∗ for aspiration and radiation shielding; ⋄ for sensors not impacted by aspiration or solar radiation shielding (e.g., sonic anemometer temperature).

### 3.2. Overview of Time Response Results

**Figure 9:**
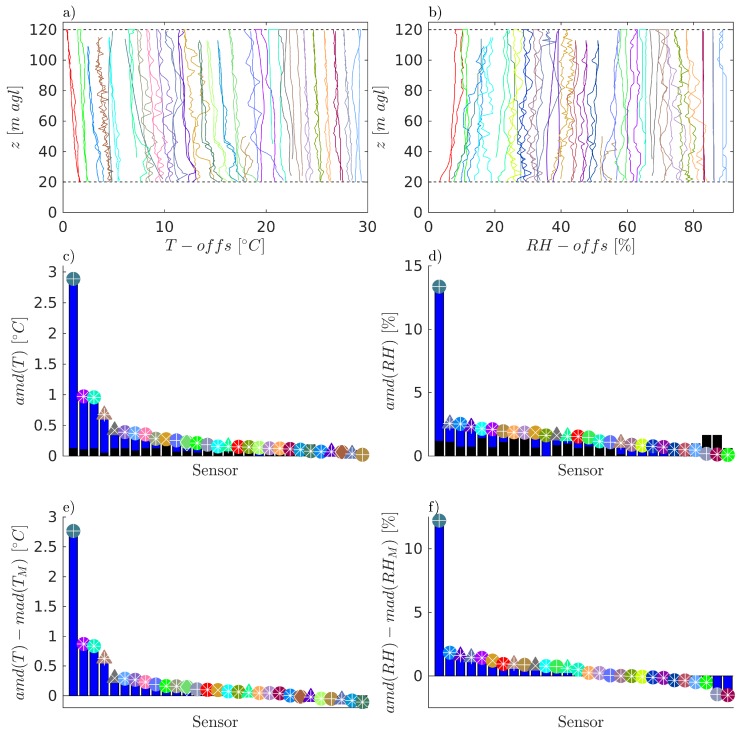
Comparison of temperature and relative humidity profiles taken during ascent and descent. Profile data between 20 m and 120 m AGL for temperature and relative humidity are shown in (**a**,**b**), respectively. The colors of the profiles are the same as in Figure 4 and Figure 5. The profile data is shifted by an artificial temperature/humidity offset to increase the visibility of the profiles. The second row shows the absolute-mean-deviation between ascent and descent data (blue) and the corresponding mean-absolute-deviation of the MURC data for the corresponding times (black) for temperature (**c**) and relative humidity (**d**). The same markers as in Figure 4 and Figure 5 are used to identify the different platform-sensor configurations. The last row shows the difference between the profile’s absolute-mean-deviation and the MURC mean-absolute-deviation for temperature (**e**) and relative humidity (**f**).

To allow assessment of the time response of the measurement systems, Figure 9 shows the results from the analysis of the difference between ascent and descent portions of the flight. We focus this comparison on the measured values of *T* and RH as these measurements are most commonly subject to slow sensor response times, and can be substantially impacted by multiple factors, including sensor type (e.g., small-bead thermistor vs integrated circuit temperature measurement) as well as the placement on the aircraft (which can impact sensor aspiration, thermal radiation from the airframe, recycling of sampled air, etc.). Hence this comparison we present is a comprehensive assessment of the entire measurement system in operation, and is important in addition to testing of sensor response time in a controlled environment.

In Figure 9a,b the *T* and RH measurements made by each sUAS during ascent and descent are compared as profiles of T(z) and RH(z) respectively, where *z* is the height AGL. To improve readability, T(z) and RH(z) have been artificially offset, with the offset varying by aircraft to minimize overlap of the profiles. The values amd(T) and amd(RH) calculated for each sUAS are presented with blue bars and with additional markers in Figure 9c,d, respectively. In Figure 9e,f, these values have been compensated by subtracting $mad(T_{M})$ and $mad(RH_{M})$ respectively, to account for non-stationarities in *T* or RH due to large-scale turbulent or synoptic scale changes that may have occurred during the flight. $mad(T_{M})$ and $mad(RH_{M})$ are also shown as black bars in Figure 9c,d. The symbol and color nomenclature used in this figure follows the one used in Figure 4 and Figure 5.

### 3.3. Temperature, Relative Humidity and Pressure

Most of the sUAS in this study carried one or more sensor payloads to measure at least three parameters: *T* (38), RH (36), and *P* (36). The intercomparisons of these parameter measurements shown in Figure 4, Figure 5 and Figure 6 indicate that the sUAS provide consistent results, with broad general agreement with the MURC reference measurements, with mean value and standard deviation of the difference between all sUAS and MURC of: *T* = 1.65 $\pm 2.6{\;}^{\circ }$C; RH = −3.15 ±12.12%; and *P* = 1.01 ±1.16 hPa. In general, higher variability of *T* measurements was reported by the sUAS as compared to the MURC during the intercomparison flight period. This result may be due to differences in sensor response times, as well as overall movement of the sUAS with respect to altitude as the sUAS often experienced altitude changes while loitering, as the mean standard deviation in altitude for all sUAS over their intercomparison time periods was 1.09 m ±0.95 m. Under well mixed conditions, resulting in a dry adiabatic lapse rate, a height difference of 1.0 m corresponds to an approximate temperature difference of 0.01 ${}^{\circ }$C at the prevailing pressure levels. It is important to note that the multirotor systems, similar to the MURC, conduct point measurements, whereas the fixed-wing aircraft were necessarily flying orbits around the MURC instruments, which can add variability due to the continually changing orientation of the aircraft.

The agreement between the sUAS and MURC mean values of measured *T* is highlighted in Figure 4d, where a perfect agreement between the two would lie along the diagonal reference line. The results indicate good agreement, with the majority (27/42) of the sUAS platforms measuring within $\pm 1{\;}^{\circ }$C of the MURC, and nearly half of the systems (11/42) measuring within $\pm 0.25{\;}^{\circ }$C. Notably, where disagreement occurred, the sUAS sensors had a positive bias, with few of the sUAS reporting mean *T* values below that of the MURC reference. In addition, these sensors tended to be of the integrated circuit-type (BME280, AQT400, MDASS). A high bias could be caused by absorption of direct solar radiation from the sun, infrared radiation from the surface, atmosphere, or warm sUAS parts (e.g., motors, battery) all of which could possibly be reduced by altering sensor placement, radiation shielding and aspiration. In at least two cases, the warmer *T* bias can be attributed to a poor sensor response (i.e., with the OSUa_SOLO_ds_10 and OSUa_SOLO_ds_13 demonstrating this in Figure 4). For these systems, the sUAS did not fully adapt to the temperature change from the warmer initial conditions close to ground to the cooler ambient air at the target loiter altitude, and we therefore did not include them in the assessment of sensor configuration.

**Figure 10:**
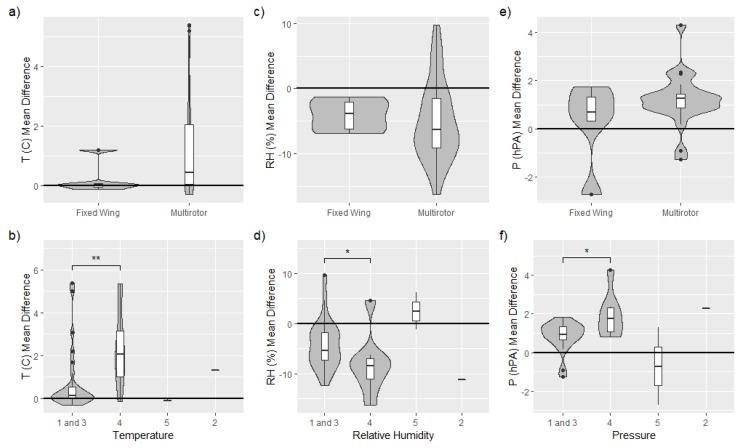
Comparison of temperature, relative humidity, and pressure measurement differences in relation to the MURC measurements, represented with the black line at 0. Averages of parameter measurement differences over each platform’s MURC intercomparison loiter period are compared between platform groups (**a**,**c**,**e**) and sensor configuration groups (**b**,**d**,**f**). For sensor configurations, 1 and 3 are sensors not impacted by solar radiation shielding or aspiration (1) or sensors with both solar radiation shielding and aspiration (3), 4 are sensors with solar radiation shielding only, 5 are sensors with aspiration only, and 2 are sensors with neither solar radiation shielding or aspiration. Wilcoxon rank-sum tests were performed (p(T)=0.009; p(RH)=0.039; p(P)=0.036).

The differences between fixed-wing and multirotor aircraft configuration are detailed further in Figure 10a, which shows the larger spread in *T* measurements from multirotors, with a positive bias predominantly measured by multirotors. When examined in further detail, by delineating whether the sensors were aspirated and solar shielded, as done in Figure 10b, a potential source of the bias emerges. *T* measurements from unaspirated sUAS sensors were significantly different than from aspirated ones (*p* < 0.009), and had a high positive bias compared to the reference. In addition, as noted above and although exceptions occurred, sUAS sensor payloads generally biasing high also largely utilized integrated circuit temperature sensing, with the small-bead thermistor and sonic anemometer sensors being in better agreement with the reference.

The sUAS and MURC intercomparison of measured RH, shown in Figure 5, indicates much less agreement between the sUAS and MURC than was observed for temperature. The same general degree of fluctuations as for *T* can be observed in the time series of RH in Figure 5a, with some systems showing more or less fluctuation in value, and nearly all reached a steady state value during the loiter period. No trend was observed in the fluctuations, potentially indicating that the different amount of RH variations observed was due to intrinsic differences between system configurations. It is clear from Figure 5d that the majority of the sUAS sensor payloads report RH mean values below that of the MURC, as mean value measurements lying along the diagonal reference line indicate perfect agreement between the MURC and sUAS, as with Figure 4d. However, only 9 of the 37 sUAS systems report RH mean values within ±5% of the MURC mean value, with most of these measured when RH<40%. Twenty sUAS systems reported mean RH values 5% to 10% lower than the MURC, in the range of RH 45% to 65%, with one system as a clear outlier measuring almost 40% higher than the MURC. The remaining four sUAS systems reported mean RH values >10% lower than the MURC, at the highest and lowest extents of the RH range.

The differences between fixed-wing and multirotor aircraft configuration are compared in Figure 10c for RH. The multirotor platforms demonstrated more variability than the fixed-wing platforms for this measurement parameter, although substantially more aircraft fall into this category. When the distributions for different sensor configurations are compared, as done in Figure 10d, it becomes apparent that much of this variability can be attributed to platforms without aspiration, with almost all platforms measuring RH mean values over 10% below that of the MURC having no aspiration. However, the measured value of RH is dependent on the measured value of *T*, and therefore the similar influence of sensor aspiration on both parameters is not unexpected.

The sUAS and MURC intercomparison of measured *P*, shown in Figure 6, indicates generally good agreement, with 24 of the 33 sUAS payload systems reporting mean values within ±1.5 hPa of the MURC reference, with all but three of those biasing higher than the MURC. There was considerable variation for the other 9 sUAS payload systems; although, all but two were within 3 hPa and most showed a positive bias as compared with the MURC reference. As shown in Figure 10e and consistent with *T* and RH, there were no statistically significant differences between *P* measurements between sUAS platforms. Similarly, the sensor configuration of *T* and RH sensors (which are commonly packaged with the *P* sensor) did have a significant impact on the measurement of *P*, as indicated in Figure 10f. Further, the sensor payloads that were placed under the body of the multirotor reported the greatest deviation from the MURC reference value and outside the range of manufacturer uncertainty for this quantity. Outside of this observation, and noting that the same types of sensors can produce different amounts of fluctuation in the measurement of *P*, the observed variability may be attributed to differences in individual sensor manufacture and intrinsic sensor properties. However, unlike RH, the difference between MURC and sUAS measurements are predominantly within the uncertainty of the majority of sensors used.

The ultimate drivers of these differences in sUAS *T*, RH, and *P* measurement agreement relative to the MURC reference measurements may be difficult to untangle. The variability in the sensor measurements over the intercomparison loiter period and the variability in the altitude may have a joint effect. The degree of variability for both *T* and RH measurements during the intercomparison loiter varies between platform and sensor configuration, and no clear trend was observed in the standard deviation of the measurements. There is no clear trend that might indicate the source of disagreement, with no observed dependence on platform type or sensor. That said, the unaspirated sensors generally reported lower values of RH, and higher values of *T*. However, this finding is not exclusive to the aspiration. Furthermore, one sensor that reported a high positive bias relative to the MURC in RH was one that had no solar shielding, although the temperature measurement, which should be most significantly impacted by solar shielding, was in close agreement for this system. An additional factor not controlled for in Figure 10 is the type of sensor itself (e.g., integrated circuit or small-bead thermistor temperature sensing, resistive or capacitative humidity sensing, etc.). Among the possible influences that sensor type could have include, susceptibility to self-heating, sensitivity to location on the aircraft (e.g., proximity to electrical noise or thermal sources), and temporal response of the sensor.

### 3.4. Wind Speed and Direction

As a vector quantity that is influenced by the motion of the sUAS, the measurement of wind speed and direction tends to require more sophisticated measurement systems. Hence, fewer systems (13) were capable of measuring wind velocity and direction as compared to the number of systems capable of measuring pressure, temperature and humidity (36). These 13 systems utilized only three types of measurement approaches, with multi-hole probes used by fixed-wing sUAS (4), sonic anemometers used by multirotor sUAS (4), and aircraft dynamics used by both types of systems (5). The last type can be further divided into three different approaches: (i) a comparison between the ground speed vector and the attitude measured by the global navigation satellite system (GNSS) and the inertial measurement unit (IMU) and the air speed, as used for the CU_DH fixed-wing sUAS; (ii) a calibrated conversion of the multirotor attitude angles for wind speed and direction while keeping the aircraft level with respect to its roll axis by yawing into the wind, as used for the three OU_CS systems; and (iii) a calibrated conversion of motor response to wind speed and direction, as used by the VT_UVA_SOLO multirotor.

Winds during the intercomparison measurements were consistent with super-adiabatic conditions at the MURC location (thereby lowering the sensitivity to altitude variability due to a well-mixed boundary layer minimizing the vertical gradient of the mean wind), with wind speeds between 2 to 4 m/s, gusting ±1 m/s over the period of each flight. In general, all measured winds showed good agreement between the MURC and sUAS, as evidenced in the time series of Figure 7a,b and scatter plot of Figure 7d. The difference in average *U* measured by all sUAS and the MURC was 0.22 ±0.59 m/s. Furthermore, the fluctuations measured by the systems were generally in good agreement as well, although some of the fixed-wing aircraft systems report higher variability than the MURC within the same time period.

**Figure 11:**
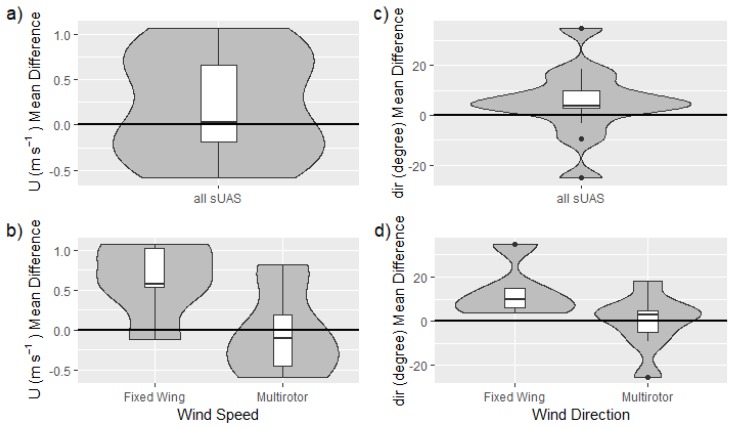
Comparison of difference (MURC – sUAS) in wind speed and wind direction measurement in relation to the MURC measurements—represented with the black line at 0. Averages of parameter measurement differences over each platform’s MURC intercomparison loiter period are shown for all sUAS and then compared by platform. Wilcoxon rank-sum tests were performed, no significant differences were reported although near-significant differences were observed: p(U)=0.09; p(dir)=0.07.

A more quantitative compilation of the differences between the sUAS and MURC is provided in Figure 11a, which shows that the sUAS were generally in close agreement with the MURC with respect to mean wind speed measured during the loiter period. A comparison of the measured mean and fluctuating velocity magnitude shows no obvious bias between sUAS and MURC measurements. The majority of the mean values measured by the sUAS are within ±0.5 m/s of the MURC mean value with a predominantly positive bias, as evident in Figure 11a.

A slightly more nuanced view is presented in Figure 11b, which divides the comparison into fixed-wing and multirotor platforms. Higher variability was reported in the fixed-wing systems, which tended to bias high. The difference in sampling flights has already been noted (e.g., multirotor systems conduct point measurements, whereas the fixed-wing aircraft fly orbits around reference instruments) but it is important to highlight that a time series measured along a horizontal circular trajectory adds variability in both wind speed and direction due to the continually changing orientation of the aircraft during each orbit, and potential horizontal inhomogeneities in the wind field. The degree of bias appears to be dependent on specifics of each aircraft, as three identical physical systems (the BC5# systems) produced bias relative to the MURC of +0.5 m/s, +0.5 m/s, and +1 m/s, suggesting potential sensitivity to the individual multi-hole probe calibrations and alignment.

The sonic anemometers on the hovering multirotors provided the most consistent results with only small deviations from the MURC values (Figure 7d). This result suggests that concerns about biasing sonic anemometer wind measurements by rotorwash can be alleviated by careful placement of the sonic anemometer, at least for mean horizontal winds. The highest variability was observed in the systems using aircraft dynamics to extract the wind velocity. Again, individual calibrations may play a role in the accuracy of the reported wind magnitude, as three identical systems reported biases relative to the MURC of −0.2 m/s, −0.5 m/s, and −0.5 m/s.

The wind direction comparison, presented in Figure 8, shows similar trends to those observed for wind speed, with an average difference between all sUAS and the MURC of 5.09 $\pm 14.07^{\circ }$. The time series comparison in Figure 8a again shows higher fluctuations in time for the fixed-wing aircraft compared to the multirotor aircraft, with these additional fluctuations attributed to the necessity of the aircraft to orbit around the MURC tower.

The distributions of average measured wind direction in Figure 11c show a tendency to measure a slightly larger angle in wind direction relative to that reported by the MURC. However, the majority of the sUAS systems measured wind direction within $\pm 15^{\circ }$ of the MURC value. As with wind speed, the distribution for all sUAS was divided into separate fixed-wing and multirotor distributions in Figure 11d. Here it can be observed that the multirotor aircraft had better agreement than the fixed-wing aircraft. Again, it is possible that the orbits flown by the fixed-wing aircraft contribute to the bias and higher variability observed in the measured wind direction.

### 3.5. Time Response of Measurement Systems

The comparison in Figure 9 provides an opportunity to assess the impact of the different sensor configurations on the time response of the temperature and humidity measurements. Note that this assessment does not take into account all possible influencing factors and is an attempt to quantify some of the effects of time response qualitatively observed when examining the data. In addition, we aim to assess the system as a whole (sensors and their arrangement on the aircraft), with the expectation that this integrated response will be quite different from manufacturer stated response.

In Figure 9c,d the higher the amd, the greater the mean difference between the ascent and descent measured profiles shown in Figure 9a,b, respectively. Assuming a stationary profile of *T* or RH, this would reflect slower response of the measurement systems. To account for non-stationarity of the true profile, these results are presented in Figure 9e,f compensated for the variability during the measurement using the mad in time reported by the MURC. This step implies the assumption that the variability observed at the height of the MURC platform is representative for the variability of the vertical column that has been profiled. It should also be noted that this analysis will not highlight systems with response times on the order of the ascent/descent times of the flight, as these systems will report the same values for both ascent and descent. Similarly, a very uniform profile, without any significant vertical gradients, is also likely to be reflected in a very good agreement between the ascent and descent profiles.

Generally, no clear trends were evident, with identical systems presenting different values. However, it does appear that the fine-bead thermistor-based temperature measurements (e.g., _xq, _xq2, and _pt100), which have faster manufacturer-stated response times, slightly outperformed the integrated-circuit-based temperature sensors (e.g., _BME280, _AQT400, _SHT31, _ds). In addition, systems with no aspiration demonstrated greater symptoms of sensor lag than those incorporating similar sensors without some form of aspiration. For example, the system that produced the largest amd(T) and amd(RH) values had the sensors mounted underneath the body of the multirotor airframe and out of the rotorwash. The systems that provided values of $amd\left(T\right)-mad\left(T_{M}\right)$ and $amd\left(RH\right)-mad\left(RH_{M}\right)$ near zero were predominantly those with some sort of aspiration of the sensors. In addition, it was observed that sUAS-sensor combinations with high negative $amd\left(T\right)-mad\left(T_{M}\right)$ and $amd\left(RH\right)-mad\left(RH_{M}\right)$ values did not seem to resolve temporal and spatial variations well (see the very straight relative humidity profiles in Figure 9b).

### 3.6. Trends and Broader Discussion

In summary, the sUAS systems show broad agreement with the MURC values, particularly for temperature. There were no clear differences between fixed-wing and mutirotor platforms in the measurement of *P*, *T*, and RH, although wind measurements made by hovering multirotor aircraft were found to be in closer agreement to the MURC measured wind speed and direction than those made by orbiting fixed-wing platforms. Variability between the wind measured by nearly identical different aircraft also suggests that individual aircraft calibrations may play a considerable role in the accuracy of these measurements.

The time response and overall accuracy of *T* and RH measurements is dependent on the aspiration and solar shielding of the sensors, which is consistent with previous work [3]. Some differences were also observed between systems using integrated-circuit-based temperature sensors and those utilizing fine-bead thermistor sensors. Non-aspirated and unshielded sensors generally deviated from the MURC reference value to a greater degree, and unaspirated sensors demonstrated symptoms of measurement lag, particularly for *T*. Bias was generally observed between the sUAS and MURC *T*, RH, and *P* measurements. The sUAS measurements were, on average, higher than the MURC measurements in *T*, and *P*, with the sUAS measurement values of RH generally lower than the MURC value. Further, the RH disagreement exceeds the stated uncertainty for the sensors used. Hence, some caution is advised when interpreting reported accuracy of RH sensors when deployed on sUAS. It should be mentioned that these biases are consistent with the sUAS being slightly lower in altitude than the MURC sensors.

## 4. Limitations and Future Work

### 4.1. Measurement Accuracy

Understanding the potential accuracy limitations of altitude measurements is particularly critical for proper assessment of all other parameters, and altitude is not trivial to measure accurately. Depending on the system, altitude is based either on GNSS or pressure alone, or a fusion of both e.g., by Kalman filtering. The corresponding altitude estimates provided by the various autopilot systems are thus prone to different types of uncertainties that might be of particular importance for the required high precision of altitude attribution in the atmospheric surface layer. The experience during the LAPSE-RATE intercomparison flights, mainly based on the mean deviation in altitude for all sUAS, suggests that some platforms had difficulties maintaining stability at the loiter height. While this was particularly true for sUAS platforms where pilots were using manual controls, sensor drift is a known challenge for sUAS altitude measurements more broadly [60].

Opportunities may exist to better capture altitude for more accurate intercomparisons with ground-based systems for future studies. For platforms and sensors where altitude is determined based on pressure, an important practice would be to validate *P* readings from sUAS simultaneously with ground-based systems while platforms are on the ground before and after intercomparison flights. These simultaneous measurements could provide a better assessment of the deviations in altitude readings when platforms are airborne and thereby may have helped to improve assessment of other atmospheric measurements. Further, we highly recommend to post-process the altitude based on the measured temperature and pressure profile if ambient atmospheric conditions are significantly deviating from the assumed temperature profile (e.g., the often assumed International Civil Aviation Organization (ICAO) Standard atmosphere is characterized by a surface temperature of 15 ${}^{\circ }$C and a lapse rate of −6.5 K/km), and are used for the pressure to altitude conversion. For low-altitude flights, a laser altimeter may help, and some of the platforms were equipped with these during the intercomparison flights (e.g., UKY’s fixed-wing sUAS). While laser altimeters are currently used to enable more precise autonomous landing (e.g., [61]), they present an opportunity for future research and development for measuring altitude for low-altitude flights. Further, a more robust assessment of dual GNSS/INS systems, GNSS systems operating under real-time kinematic positioning, and magnetometer drift could aid in better atmospheric measurements as sUAS altitude and orientation is critical for accurate sensor intercomparison measurements.

A response rate comparison with the sUAS sensor measurements compared with the MURC measurement was intended using information gathered as the sUAS platform passed through the MURC point of altitude on ascent and descent, however, we were not able to conduct detailed analysis using this data. Rigorous analysis of the sensor response time using flight data was inhibited by the well-mixed prevailing conditions during the intercomparison flights causing weak vertical gradients. Detailed analysis of time response of the measurement systems using this portion of the flights is therefore not possible in this study, but is an important area for future research. Related to response time, for the intercomparison loiter period, measurements were used when the aircraft was determined to be at the appropriate loiter altitude and where sensor readings appeared to reach a measurement equilibrium. Further characterization and assessment of the time for sensor systems to reach the measurement equilibrium upon reaching the loiter altitude may provide useful insights for developing the best flight plans for atmospheric measurement data capture.

An important practice to explore for future intercomparison studies would be to include comparisons of the atmospheric measurements between all sensors while the sUAS are on the ground, and to do so under a variety of meteorological conditions. This could provide a useful baseline for assessing the accuracy and robustness of sensor measurements in flight. Further, future intercomparison studies may be able to determine a calibration factor that could be used based on the reference data in order to make the measurement data from all sUAS platforms align with the reference system. This would be helpful to a research study or operations team aiming to use data from different platforms collectively to address broader scientific inquiries. However, with the current intercomarison data, we are unable to make this described calibration factor. To do so in the future, intercomparison flights would need to be conducted in a variety of atmospheric conditions (e.g., at night with no solar radiation) and repeated more than once in order to determine a robust calibration factor. One important limitation to note is the lack of robust validation of the MURC reference measurements. While MURC instrumentation was calibrated as described in Section 2, and radiosondes and other mobile ground-based systems (e.g., CoMeT) provided important contextual comparison measurements, more comprehensive validation of the MURC reference measurements may have benefited this intercomparison analysis.

### 4.2. Measurement Robustness

Addressing temporal drift of intercomparison results (e.g., comparing intercomparison flights between the first day and final day of the week-long LAPSE-RATE campaign) is another important consideration. Comparing these same sUAS sensor payloads over the course of the longer campaign would allow for important discussions of the affect of ‘wear and tear’ of sensor systems on measurement accuracy. It is critical to extend the assessment of these sensor systems beyond laboratory or ground-based conditions, to more comprehensively evaluate sensor robustness under flight operation conditions. While we did attempt an intercomparison event on the final day of the LAPSE-RATE campaign (20 July 2018), operational challenges prevented several teams from participating. Flight tests conducted on this final day demonstrated the utility of calibrating multiple sensors on a single platform, providing improved confidence (or at least understanding) of intercomparisons between sensor systems. Even in these cases, however, mounting on small platforms may introduce discrepancies due to differences in aspiration and radiation shielding.

Additionally, as sensor payloads are occasionally exchanged between platforms during longer campaigns, it may also be beneficial to conduct intercomparison flights with the same sensor payloads flown on different aircraft frames. Examples of calibration experiments to evaluate for characterizing sensor response have been addressed elsewhere, primarily by utilizing simple calibration and validation experiments to determine the impact of response times and drift over limited times [3]. However, longer-term experiments are needed to better understand the impact of sensor behavior and associated measurement confidence. Utilization of Observational System Simulation Experiments (OSSEs) is one way, as it provides the ability to investigate the optimal observational sUAS deployment and sensor requirements in a simulated setting, including number and spatial distribution of weather observations, cadence of the measurements, spatial and temporal resolution, and vertical sampling resolution, for example [62,63,64].

### 4.3. Data and Information Management

There are many other potential sources and drivers of measurement error to consider. Additional sensor configuration considerations are likely relevant beyond solar radiation shielding and aspiration, and more comprehensive assessment of instrumentation handling is worth highlighting for future work (e.g., internal heating of sensors, as sUAS sensors measuring higher *T* and lower RH than the MURC, which can potentially be due to internal heating caused by their locations and configurations within the sUAS). Further, instrument calibration methodology, data corrections, and post-processing differ by sensor, system, and operator. To evaluate sensor system performance, there is great need to better represent the data processing levels involved (e.g., sensor manufacturer corrections, post-processing corrections) and whether intercomparison assessments are occurring with minimum data post-processing, or to what extent data are subjected to post-processing routines including quality assurance and quality control routines.

All sUAS platforms in this study had varying instrument handling and data management processes and workflows, with differing levels of automation regarding on-board data processing and post-processing. All together, this auxiliary information is likely critical for characterizing sUAS measurement accuracy, but is often difficult to capture as usable metadata, as operators may have a variety of different methods and expectations for documenting this information. However, it is clear that capturing relevant metadata in more detail and in a standardized way is critical to better assess sUAS measurement accuracy, error, and uncertainty and to improve future scientific goals (e.g., integrating sUAS data into weather models). To advance the use of sUAS for scientific data collection, future work will need to address these and other relevant data and information management challenges.

## 5. Conclusions

As small unmanned aircraft become more prominent platforms for atmospheric data collection, this paper highlights results from one of the largest collaborative flight campaigns with in-field intercomparison measurements to date, with a wide variety of differing platforms, sensors, and data collection workflows. We demonstrate the important role of coordinated intercomparison flights to support and enable the overall progression of sUAS collecting scientific measurements of atmospheric parameters. In particular, we show that while sUAS are broadly providing accurate atmospheric measurements, sensor configuration is important and proper aspiration and solar radiation shielding are likely more important than sUAS platform or specific sensor type. These significant differences highlight the need for more comprehensive sUAS design and development, with careful consideration for sensor integration to minimize measurement accuracy differences between platforms and enable more robust atmospheric data collection. Important challenges we highlight are managing all of the scientifically relevant data from the sUAS and capturing the specifics of important data-collection operations details (e.g., sensor specifications and configurations, instrument calibration, and data-processing workflows). Overall, we conclude that the future is bright for the use of sUAS for atmospheric science and expanding our understanding of best-practices in sUAS data collection via this and future intercomparision studies will lead to increasing the usefulness for sUAS-collected data in augmenting and expanding atmospheric knowledge, and for the potential for integrating these data for broader scientific insights.

## Figures and Tables

**Table 1:** Aircraft participating in intercomparison experiment.Operators were: CU, University of Colorado-Boulder; CU-BST, University of Colorado-Boulder and Black Swift Technologies; EGM, EngeniusMicro LLC; FMI, Finnish Meteorological Institute; KSU, Kansas State University; OSU-A, Oklahoma State University Team A; OSU-B, Oklahoma State University Team B; OSU-C, Oklahoma State University Team C; OU, University of Oklahoma; UNL, University of Nebraska–Lincoln; UKY, University of Kentucky; VT, Virginia Tech; UVA, University of Virginia. Nomenclature in table includes: R, multirotor; FW, fixed-wing; *P*, pressure; *T*, temperature; RH humidity; *U*, wind velocity; MHP, multi-hole probe. Configuration types refer to the configuration of the temperature and humidity sensors and are: 1—sensor packages where aspiration and solar shielding are not relevant; 2—no special sensor placement, aspiration, radiation protection; 3—sensors have aspiration and solar shielding; 4—sensors have solar shielding but no forced aspiration; 5—sensors have aspiration but no solar shielding; *—indicates data not available at time of manuscript preparation; #—indicates where numbers used in figure legends to denote different aircraft or sensors as appropriate.

Operator	Airframe	Type	QTY	Measures	Sensor				Config.	Legend
					P	RH	T	U		
CU	Data Hawk2	FW	3	PTHU		Sensiron SHT-31			3	CU_DH_SHT31
					TE MS-5611					CU_DH_MS5611
							Cold-wire		3 *	
								Aircraft Dynamics		CU_DH_IMU
	Mistral	FW	1	PTHU	BST MHP				5 *	
	TTwistor	FW	1	PTHU	Vaisala PTU module				3 *	
								Aeroprobe MHP	*	
	Talon-3	FW	1	PTH	Vaisala PTU module				3 *	
					TE MS8607				*	
CU-BST	S1	FW	1	PTHU	BST MHP				5	CU_S1_MHP
	S2	FW	1	PTHU	BST MHP				5 *	
EGM	Intense Eye V2	R	1	PTHU			DTS Flex (×2)		4	EGIM_IE2_DTS_#
					TriSonica Mini WS				1	EGM_IE2_tris
FMI	Tarot Hex	R	2	PTH	Bosch BME 280				4	FMI_P#_BME280
					Vaisala AQT400				4	FMI_P2_AQT400
KSU	M600	R	1	PTH	OSU MDASS				3	KSU_M600_ds_09
OSU-A, OSU-C	SOLO	R	6	PTH	OSU MDASS				3	OSU#_SOLO_ds_##
OSU-A	M600	R	1	PTHU	iMetXQ2				2	OSUa_M600P1_xq2
					OSU MDASS				3 *	
							FT205		1 *	
	M600	R	1	PTHU	OSU MDASS				3 *	
							Young 81000		1	OSUa_M600P2_Y81
OSU-B	SOLO	R	3	PTH	iMet XQ2				4	OSU_SOLO_xq2_#
OU	Coptersonde 2	R	3	PTHU	TE MS5611					OU_CS2#_MS5611
						IS HYT271			4	OU_CS2#_HYT271_#
							iMet Therm. (×3)		3	OU_CS2#_PT100_#
								Aircraft Dynamics		OU_CS2#_IMU
UKY	M600	R	1	PTH	iMetXQ2 (×2)				3	UKY_M600P_xq2_#
	SOLO	R	1	PTHU	iMetXQ2				3	UKY_SOLO_xq2
							TriSonica Mini		1	UKY_SOLO_tris
	SOLO	R	1	PTH	Bosch BME 280				4	UKY_SOLO_BME280
	S1000	R	1	PTH	iMetXQ				3	UKY_S1000_xq1
	BLUECAT5	FW	3	PTHU	iMetXQ				3	UKY_BC5#_xq1
								Custom MHP		UKY_BC5#_MHP
UNL	M600	R	1	PTH	iMetXQ				3	UNL_M600P1_xq1
					iMetXQ2				3	UNL_M600P1_xq2
	M600	R	1	PTH	iMetXQ2				3	UNL_M600P2_xq2
					Nimbus PTH				3	UNL_M600P2_nim_1
					Nimbus PTH				4	UNL_M600P2_nim_2
VT, VT-UVA	SOLO	R	1	PTHU	iMet XQ			Aircraft Dynamics	5	VT_UVA_SOLO_xq2
	Inspire 2	R	2	TU			Meter Atmos 22		1	VT_I2#_MA22

## Abbreviations

The following abbreviations are used in this manuscript:

AGL	Above Ground Level
CLAMPS	Collaborative Lower Atmospheric Mobile Profiling System
COA	Certificate of Authorization
COST	European Cooperation in Science and Technology
COTS	commercial off the shelf
FAA	Federal Aviation Administration
GNSS	Global Navigation Satellite System
ICAO	International Civil Aviation Organization
IMU	Inertial Measurement Unit
INU	Inertial Navigation Unit
IRISS	Integrated Remote and In-Situ Sensing program
ISARRA	International Society for Atmospheric Research using Remotely piloted Aircraft
LAPSE-RATE	Lower Atmospheric Process Studies at Elevation—a Remotely piloted Aircraft Team Experiment
MDT	Mountain Daylight Time
MSL	Meters above Sea Level
MURC	Mobile UAS Research Collaboratory
NOAA	National Oceanic and Atmospheric Administration
NSSL	NOAA National Severe Storms Laboratory
OSSEs	Observational System Simulation Experiments
RPAS	Remotely Piloted Aircraft System
sUAS	small Unmanned Aircraft System
